# Directly Bound Deuterons Increase X‐Nuclei Hyperpolarization using Dynamic Nuclear Polarization

**DOI:** 10.1002/cphc.202300144

**Published:** 2023-07-24

**Authors:** Catriona H. E. Rooney, Ayelet Gamliel, David Shaul, Damian J. Tyler, James T. Grist, Rachel Katz‐Brull

**Affiliations:** ^1^ Department of Radiology Hadassah Medical Organization and Faculty of Medicine Hebrew University of Jerusalem Jerusalem 9112011 Israel; ^2^ Department of Physiology Anatomy and Genetics University of Oxford Oxford UK; ^3^ The Wohl Institute for Translational Medicine Hadassah Medical Organization Jerusalem Israel; ^4^ Oxford Centre for Clinical Magnetic Resonance Research Division of Cardiovascular Medicine Radcliffe Department of Medicine University of Oxford Oxford UK; ^5^ Department of Radiology Oxford University Hospitals Oxford UK; ^6^ Department of Biomedical and Neuromotor Sciences University of Bologna Bologna Italy

**Keywords:** D-glucose, 2-deoxy-D-glucose, solid-state polarization, urea, sodium nitrate

## Abstract

Deuterated ^13^C sites in sugars (D‐glucose and 2‐deoxy‐D‐glucose) showed 6.3‐to‐17.5‐fold higher solid‐state dynamic nuclear polarization (DNP) levels than their respective protonated sites at 3.35T. This effect was found to be unrelated to the protonation of the bath. Deuterated ^15^N in sites bound to exchangeable protons ([^15^N_2_]urea) showed a 1.3‐fold higher polarization than their respective protonated sites at the same magnetic field. This relatively smaller effect was attributed to incomplete deuteration of the ^15^N sites due to the solvent mixture. For a ^15^N site that is not bound to protons or deuterons ([^15^N]nitrate), deuteration of the bath did not affect the polarization level. These findings suggest a phenomenon related to DNP of X‐nuclei directly bound to deuteron(s) as opposed to proton(s). It appears that direct binding to deuterons increases the solid‐state DNP polarization level of X‐nuclei which are otherwise bound to protons.

## Graphical abstract

**Deuterated** ^**13**^ **C sites** show 6.3‐to‐17.5‐fold higher solid‐state dynamic nuclear polarization (DNP) induced hyperpolarization than their respective protonated sites. Deuterated ^15^N sites show 1.3‐fold higher polarization than their respective protonated sites. These findings suggest the utility of deuteration, where possible, for increased polarization level in solid‐state.

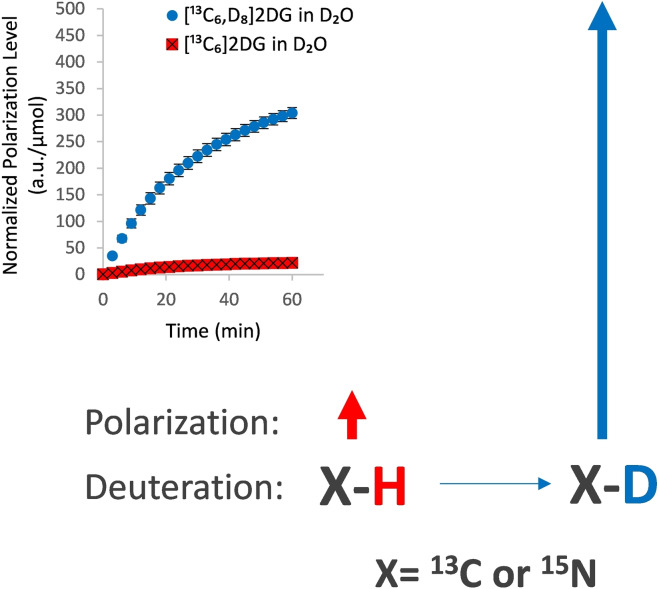

## Introduction

The process of ^13^C polarization enhancement in the solid‐state under dynamic nuclear polarization (DNP) conditions (<40 K, in the presence of free radicals and microwave (MW) irradiation( consists of multiple mechanisms, the different contributions from which are condition and formulation dependent.^[1]^ This means that the combination of the molecule bearing the ^13^C nuclei, the vitrifying solution, the radical, the temperature, and the irradiation frequency are all part of this process, and each may affect the polarization buildup time, the T_1_ in the solid‐state, and the maximal polarization that may be achieved.

Previously, the factors influencing this process in several compounds and formulations involving ^13^C hyperpolarization in the solid‐state were characterized.^[2]^ For biomedical applications, the utility of ^13^C‐labeled compounds for dissolution DNP (dDNP) studies is of interest. The dDNP approach allows enhancement of the liquid‐state polarization of ^13^C sites by >10,000 fold^[1a]^ and thus allows monitoring of metabolic processes in biological preparations and *in vivo* in human subjects in real time.^[3]^ Specifically, studies have been focused on ^13^C sites that are directly bound to deuterons (D), as a means to prolong the ^13^C T_1_ in solution, in sites that would otherwise (*i. e*., when protonated) be inaccessible to dDNP due to the fast decay of their hyperpolarized state in solution (a few seconds).[^2^, ^4^] The utility of agents of metabolic potential such as choline, D‐glucose, and 2‐deoxy‐D‐glucose, that are doubly labeled in this way, *i. e*. where ^13^C sites are directly bound to deuterons, has been previously shown.^[5]^

Our recent study on the effect of Gd^3+^ doping on [^13^C_6_, D_7_]glucose polarization^[2]^ raised the question of whether the direct binding of deuterons to the ^13^C sites (as opposed to ^13^C‐^1^H bonds) also affects the polarization buildup process.^[2]^ Deuterium atoms are chemically equivalent to hydrogen atoms but weigh twice as much and are larger (0.8768(69) fm for proton *vs*. 2.12562(78) fm for deuteron^[6]^). The C−H bond in sp^3^ hybridized carbon is 1.091–1.094 Å^[7]^ and the C−D bond is about 0.005 Å shorter.^[8]^ These differences are part of what is known as the “isotopic effect” which has been affiliated with differences in reactivity and pharmaceutical effects that occur upon deuteration of one or more sites in certain compounds.^[9]^ It is important to note, however, that the metabolism of glucose by glycolysis does not appear to be perturbed due to deuteration.^[10]^

The ^13^C nano environment, including the identity of the magnetically active nuclei in that nano environment, could, in principle, affect the DNP process of the ^13^C sites. For this reason, and due to the importance of deuterated compounds as potential new dDNP molecular imaging agents, we set out to investigate whether deuteration affects the polarization process in the solid‐state.

We present a study of two sugar molecules, D‐glucose (Glc) and 2‐deoxy‐D‐glucose (2DG). Analogs of both these compounds, doubly labeled with ^13^C and D, have been previously studied with dDNP.[^5b^, ^5d^, ^11^] Two stable‐isotope‐labelling schemes were used to answer the above question for ^13^C sites: 1) sugars uniformly labeled with ^13^C and 2) sugars uniformly doubly labeled with ^13^C and D. In the former case, all ^13^C sites were directly bound to one or two protons (as per the specific site). In the latter, each ^13^C site was directly bound to one or two deuterons (as per the specific site) and no ^13^C sites were directly bound to protons. In addition, to study this potential influence comprehensively, we have also studied the effect of deuteration on ^15^N solid‐state polarization in model compounds such as urea and nitrate. The DNP polarization process of these protonated and deuterated analogs was recorded and compared at 3.35 T.

## Results

**Figure 1:**
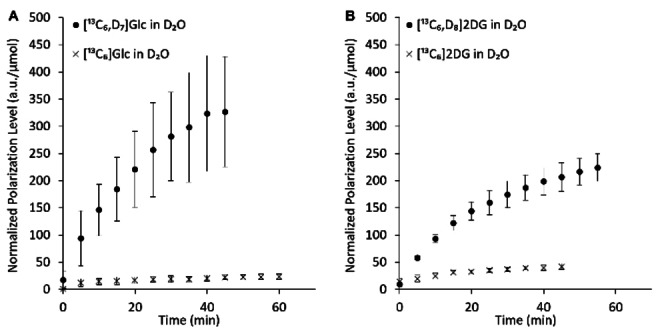
Polarization buildup for deuterated and non‐deuterated ^13^C‐uniformly‐labeled sugars. A) Glc formulations, B) 2DG formulations. The actual polarization levels in arbitrary units for each time course were corrected for the number of sugar moles in the cup. Then, the values at each time point were averaged for each formulation. The error bars represent the standard deviation for each time point. Seven buildup time courses were recorded for [^13^C_6_,D_7_]Glc in D_2_O, and three for all other formulations. The individual buildup time courses and the curve fitting for each to Eq. 1 are shown in Figure S2.

Figure 1 demonstrates the polarization buildup process for the sugar formulations under investigation (Note S1, Tables S1, S2, S3, S4, and Figure S1). The deuterated formulations reached a much higher polarization level (17.5‐fold and 6.3‐fold higher for Glc and 2DG, respectively, Table 1). The buildup time constant was prolonged for the deuterated sugars (1.8‐fold and 2.0‐fold longer for Glc and 2DG, respectively, Table 1).

**Table 1:** Polarization buildup time constants and maximal polarization levels.

Compound/Solvent/Formulation number*	Buildup time constant (min)	Maximum polarization level corrected for μmol agent (arbitrary units)	Buildup time constant prolongation** (fold)	Increase in maximal polarization** (fold)
D_2_O‐ or H_2_O‐only formulations of deuterated and non‐deuterated ^13^C‐uniformly‐labeled sugars				
[^13^C_6_,D_7_]Glc in D_2_O, #1A, n=7	24±4	403±124	1.8^a1^	17.5^a2^
[^13^C_6_,D_7_]Glc in H_2_O, #1C, n=3	22±2	337±79		
[^13^C_6_]Glc in D_2_O, #1B, n=3	13±12	23±4		
[^13^C_6_,D_8_]2DG in D_2_O, #2A, n=3	22±1	253±30	2.0^b1^	6.3^b2^
[^13^C_6_]2DG in D_2_O, #2B, n=3	11±5	40±4		

D_2_O:glycerol or H_2_O:glycerol formulations of ^15^N‐labeled compounds				
[^15^N_2_]urea in D_2_O:glycerol, #3A, n=3	14±1.4	0.67±0.06		1.3^c1^
[^15^N_2_]urea in H_2_O:glycerol, #3B, n=3	32±13	0.51±0.04		
Sodium [^15^N]nitrate in D_2_O:glycerol, #4A, n=3	67±38	0.13±0.07		1.0^d1^
[^15^N]nitrate in H_2_O:glycerol #4B, n=3	70±33	0.13±0.03		

[*] Formulation number as indicated in Note S1 and Table S1. [**] due to directly bound deuteron(s) or D2O in the polarization mixture. Values are given as average ± standard deviation (n=number of experiments). Max. polarization values of sugars do not correspond to those of the ^15^N‐labeled agents. [a1] comparing [^13^C_6_,D_7_]Glc to [^13^C_6_]Glc, both in D_2_O, p=0.06; [a2] comparing [^13^C_6_,D_7_]Glc to [^13^C_6_]Glc, both in D_2_O, p=0.0009; [b1] comparing [^13^C_6_,D_8_]2DG to [^13^C_6_]2DG, both in D_2_O, p=0.02; [b2] comparing [^13^C_6_,D_8_]2DG to [^13^C_6_]2DG, both in D_2_O, p=0.0003; [c1] comparing [^15^N_2_]urea in D_2_O and H_2_O, p=0.02; [d1] comparing [^15^N]nitrate in D_2_O and H_2_O, p=1. All comparisons were performed with a two‐tailed Student's t‐test.

**Figure 2:**
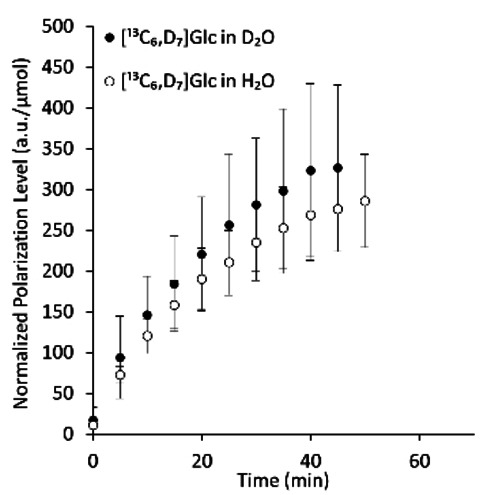
Polarization buildup for deuterated and ^13^C‐uniformly‐labeled Glc in D_2_O and H_2_O baths. The actual polarization levels in arbitrary units for each time course were corrected for the number of sugar moles in the cup. Then, the values at each time point were averaged for each formulation. The error bars represent the standard deviation for each time point. Seven buildup time courses were recorded for [^13^C_6_,D_7_]Glc in D_2_O, and three for the formulation in H_2_O. The individual buildup time courses and the curve fitting for each to Eq. 1 are shown in Figure S2. The data for [^13^C_6_,D_7_]Glc in D_2_O are the same as those shown in Figure 1A.

To assess whether the solvent spin bath plays a role in the polarization of sp^3 13^C with directly bound deuterons, we performed an additional comparison to a different formulation of [^13^C_6_,D_7_]Glc which was prepared in H_2_O (instead of D_2_O). We found that polarizing this agent in such a bath, which is rich in ^1^H, resulted in a maximal polarization level that was similar to that obtained in the D_2_O bath (Table 1 and Figure 2). This finding suggested that the results obtained in the D_2_O‐only formulations are not related to a bath effect but to the direct binding of ^13^C to D instead of to ^1^H.

To further test this phenomenon, we turned to a different X‐nucleus, namely ^15^N. As a first step in this characterization, we recorded the MW profile of [^15^N_2_]urea and [^15^N]nitrate formulations (Note S1, Table S1 and Figure S3). These profiles were found to be similar (Figure S3). The minimum peak of each MW profile was then used to monitor the buildup process of ^15^N nuclei in the polarizer.

[^15^N_2_]urea was used as a model molecule. Two formulations of [^15^N_2_]urea were prepared as described in the Note S1 and Table S1. The formulation of [^15^N_2_]urea, which was prepared in H_2_O, (formulation #3B), contained [^15^N_2_]urea without further isotopic labeling. However, the formulation which was prepared in D_2_O, (formulation #3 A), contained [^15^N_2_]urea that was also labeled with deuterium, due to the quick exchange of the exchangeable protons with D_2_O.^[12]^ However, because this formulation also contained glycerol (non‐deuterated), and the possible H−D exchange between glycerol and D_2_O, the number of deuterons per [^15^N_2_]urea is not certain.

**Figure 3:**
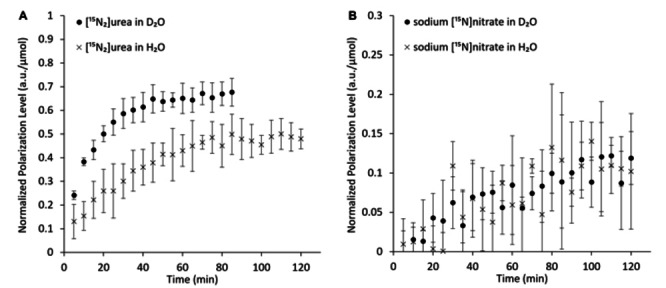
Polarization buildup for [^15^N_2_]urea and sodium [^15^N]nitrate in D_2_O:glycerol and H_2_O:glycerol formulations. A) [^15^N_2_]urea, B) sodium [^15^N]nitrate. The actual polarization levels in arbitrary units for each time course were corrected for the number of compound moles in the cup and to the number of ^15^N nuclei in each compound, *i. e*., the actual mole normalized data for [^15^N_2_]urea are 2‐fold higher than for [^15^N]nitrate. The values at each time point were averaged for each formulation. The error bars represent the standard deviation for each time point (n=3 for all). The individual buildup time courses and the curve fitting for each to Eq. 1 are shown in Figure S4.

We found that indeed, as for ^13^C, direct binding to D instead of ^1^H led to a higher polarization level for the ^15^N site (1.3‐fold, Figure 3A, and Table 1). The buildup time constant was not significantly affected by the directly bound deuterons (Table 1).

For [^15^N_2_]urea, the deuteration of the bath directly affects the binding of deuterons to the ^15^N site. To test for the possible effects of bath deuteration on ^15^N hyperpolarization *per se*, we used another model molecule, namely sodium [^15^N]nitrate, which does not have any proton binding sites. For [^15^N]nitrate, the results suggested that deuteration of the bath did not significantly change either the polarization level of the ^15^N site or the polarization buildup time constant (Figure 3 and Table 1).

This further strengthens our finding that direct deuteration of X‐nuclei benefits the polarization level of these hyperpolarized sites but nearby deuterons from the bath do not affect this process, in the conditions tested here.

## Discussion

To the best of our knowledge, this is the first solid‐state investigation concerning the effect of direct deuterium binding on the DNP process of X‐nuclei. We studied the effect on both ^13^C and ^15^N sites with both displaying higher polarization upon direct binding to deuterium atoms compared to protons. By testing these conditions in H_2_O and D_2_O baths, including in an ^15^N labeled compound that does not have any protonation sites, we have shown that the phenomenon observed here is not related to the bath but rather to the direct binding of the X‐nuclei to deuterium atom(s), as opposed to proton(s). The current finding for ^13^C and ^15^N adds to a previous report on the qualitative correlation between the DNP polarization of ^13^C and ^15^N, in [^13^C]urea and [^15^N_2_]urea, respectively.^[13]^ While the solid‐state ^13^C polarization was extensively studied, the solid‐state polarization of ^15^N is less researched. Thus, it is interesting to see that at least in two aspects, ^13^C and ^15^N share similar characteristics, *i. e*., 1) the effect of directly bound deuterium atoms, and 2) the correlation between the polarization levels of these nuclei.

The increase in the polarization level of the deuterated compounds was not expected, however, it is likely in line with the prolonged T_1_ of these sites in solid‐state (Figure S5) and in solution^[5b]^ (Table S7). The prolonged solid‐state T_1_s of the deuterated sites likely leads to favorable polarization buildup conditions. In our hands, other compounds with ^13^C that were protonated and showed short T_1_s in solution (of the order of a few seconds) did not show significant buildup in the polarizer.^[13]^ Such molecules were investigated as possible ^13^C solid‐state markers for polarization of other X‐nuclei in the same polarizer and include, for example, [2‐^13^C]glycerol and [^13^C_3_]glycerol.^[13]^ Formulations of these compounds failed to produce detectable ^13^C solid‐state polarization in less than 15 min and less than 20 mg formulation.^[13]^

Previously, Niedbalski *et al*.^[14]^ reported that deuteration of an sp^3^ carbon position slightly reduced the maximal attainable ^13^C polarization of the deuterated site and an adjacent SP^2 13^C site. This was reported for a different molecule (acetate), in a different glassing matrix (1 : 1 glycerol : water), but with the same radical (OX063, 15 mM), magnetic field, and polarization temperature (1.4 K)^[14]^ as in the current study. Interestingly, in the same study, the authors found that the maximal attainable ^13^C polarization was correlated to the T_1_ of that site in the solid‐state at the same temperature. The difference in site and glassing matrix is the most likely cause for the different results obtained in the current study for ^13^C.

Indeed, the effect of the matrix deuteration was previously investigated in water‐alcohol mixtures,^[15]^ in glycerol:water and DMSO:water mixtures,^[16]^ and in other mixtures of organic solvents.^[16b]^ It was previously reported that for the radical used here (OX063), using a deuterated solvent led to lower polarization of the ^13^C site.^[16]^ However, the formulations used in the current study for sugar ^13^C hyperpolarization were different, as they were solely aqueous. The current sugar formulations make use of the fact that saturated or highly concentrated solutions of sugars (such as Glc and 2DG) and organic salts (such as choline) in water, vitrify in cryogenic temperatures and do not require the presence of organic vitrification agents such as glycerol or DMSO. In fact, Glc and other sugars are considered cryo‐protectants.^[17]^ Saturated solutions of Glc also provide benefit in terms of concentration of the agent in the formulation (a maximum of 54 g/100 ml solution in the current formulations (Glc to total solution volume, see Table S5, mixture number 1) compared to *ca*. 48 g/100 ml solution, in a 50 : 50 water:glycerol solution^[18]^). Such formulations were previously used for the study of choline, Glc, and 2DG analogs in a hyperpolarized state.[^4a^, ^5a^, ^5b^, ^5c^, ^19^] We could not find a previous study of the deuteration of the matrix in such formulations.

We note that the current study on sugar formulations was carried out in formulations doped with Gd^3+^. This was done in agreement with a previous optimization study of Gd^3+^ in Glc hyperpolarization.^[2]^ The beneficial effect of Gd^3+^ doping for polarization of ^13^C sites agreed with prior reports on formulations that used organic:water mixtures as solvent and the same radical (OX063).[^16c^, ^20^] The formulations used here have previously shown 13.2±4.8 % polarization in solution .^[5d]^

Table S6 summarizes the various conditions of protonation and deuteration of X‐nuclei tested in this work using model compounds. The effect of directly bound deuteron(s) on protonation sites of X‐nuclei was tested in three compounds: [^13^C_6_]Glc, [^13^C_6_]2DG, and [^15^N_2_]urea. The effect of the bath deuteration was tested in two compounds: [^13^C_6_, D_7_]Glc and sodium [^15^N]nitrate. The enhancement of maximal polarization due to deuteration of proton binding sites was much more pronounced for the ^13^C sites compared to the ^15^N site. One possible explanation for this is that the ^15^N formulations contained glycerol (non‐deuterated). Exchange between the glycerol's protons and the D_2_O deuterons could lead to less than full deuteration of [^15^N_2_]urea and in this way dilute the potential direct deuteration effect.

## Conclusions

Under the formulation and DNP polarization conditions investigated here, deuterated ^13^C sites showed 6.3‐to‐17.5‐fold higher polarization than their respective protonated sites, and ^15^N sites showed 1.3‐fold higher polarization than their respective protonated sites. This suggests that the current findings represent a phenomenon related to DNP of X‐nuclei directly bound to deuteron(s) as opposed to proton(s). It points to the utility of deuterium direct bonding in increasing the polarization level that can be achieved for molecular sites where this could be relevant. It also points to the utility of dissolving agents with exchangeable protons in D_2_O as this will in practice label the relevant X‐sites with deuterium and increase their polarizability. For example, prior to the current study it was known that dissolution in D_2_O will prolong the visibility time window of [^15^N_2_]urea,^[12]^ but it was not known that the polarizability of [^15^N_2_]urea can benefit from the addition of D_2_O into the formulation mixture.

## Experimental

Polarizations were performed in solid‐state at 1.4–1.5 K, in DNP polarizers operating at 3.35 T. Sugar formulations were prepared in water (naturally abundant or deuterated) and contained 2.1 to 2.4 μmol of the particular sugar per mg formulation, 13.3 to 14.0 mM OX063 radical and 0.87 to 0.91 mM Gd^+3^. Formulations containing ^15^N‐labeled compounds were prepared in 60 : 40 water:glycerol (naturally abundant water or deuterated water) and contained 4.68 to 5.65 μmol of the labeled compound per mg formulation and 11.5 to 14.9 mM OX863 radical. Further information is provided in the Supporting Information.

## Supporting Information

The authors have cited additional references within the Supporting Information.^[21]^

## Conflict of interest

The authors declare no conflict of interest.

1

## Supporting information

As a service to our authors and readers, this journal provides supporting information supplied by the authors. Such materials are peer reviewed and may be re‐organized for online delivery, but are not copy‐edited or typeset. Technical support issues arising from supporting information (other than missing files) should be addressed to the authors.

Supporting Information

## Data Availability

The data that support the findings of this study are available in the supplementary material of this article.
