# Supplementary material for: Directly Bound Deuterons Increase X‐Nuclei Hyperpolarization using Dynamic Nuclear Polarization
Source: Chemphyschem. 2023 Jul 24;24(18):e202300144. doi: 10.1002/cphc.202300144 (PMC10947409; doi:10.1002/cphc.202300144)
Supplement: Supplementary file 1 — Supporting Information [file CPHC-24-0-s001.pdf]

ChemPhysChem

Supporting Information

Directly Bound Deuterons Increase X-Nuclei Hyperpolarization using Dynamic Nuclear Polarization

Catriona H. E. Rooney[+], Ayelet Gamliel[+], David Shaul, Damian J. Tyler, James T. Grist[+], and Rachel Katz-Brull[+]*

Supporting information

Note S1. Materials and Methods

Chemicals

The OX063 radical (GE Healthcare, Chalfont Saint Giles, UK) was obtained from Oxford Instruments Molecular Biotools (Oxford, UK). [$^{13}C_6$]Glc was purchased from Sigma-Aldrich (Rehovot, Israel). [$^{13}C_6$]2DG was purchased from Omicron Biochemicals (South Bend, IN, USA). [$^{13}C_6$,D_7]Glc was obtained from Cambridge Isotope Laboratories (Tewksbury, MA, USA). [$^{13}C_6$,D_8]2DG was obtained from 13C Molecular (Fayetteville, NC, USA). Gd^{3+} as gadoteric acid - gadoterate meglumine was obtained from Guerbet (Dotarem, Villepinte, France). [$^{15}N_2$]urea was obtained from Cambridge Isotope Laboratories (Tewksbury, MA, USA). Sodium [^{15}N]nitrate was obtained from Sigma-Aldrich (Dorset, UK).

Spin polarization

^{13}C spin polarization was performed in a dDNP spin polarizer (HyperSense, Oxford Instruments Molecular Biotools, Oxford, UK) operating at 3.35 T. Irradiation was performed with a power of 100 mW at 1.5 K for 1.5-2 h. The polarization data were recorded using the built-in spectrometer within the polarizer. Microwave frequency was optimized for each formulation. The first maxima for each of the sugar formulations used in this study was found to be the same (Figure S1), and this frequency was used for monitoring the polarization buildup process.

^{15}N spin polarization was carried out in an alpha prototype spin polarizer (Oxford Instruments Molecular Biotools, Oxford, UK), operating at 3.35 T, and equipped with a custom-built tuning-and-matching box that enabled monitoring of ^{15}N solid-state signal using the original ^{13}C solid-state probe of the polarizer. The tuning-and-matching box consisted of a Pi-match impedance circuit made with two variable capacitors to enable operation at the desired frequency (14.45 MHz). The solid-state signal was observed on a Varian spectrometer (Agilent, Santa Clara, CA). For ^{15}N labeled compounds, polarization buildup experiments were carried out at ~1.2 K with a microwave power of 100 mW, for 1.5-2 h. Microwave frequency was optimized for each formulation.

Composition of formulations for polarization

Typical formulations for the hyperpolarization of the sugar analogs are described below and in Table S1. The concentration of the sugar in the formulation was calculated by dividing the number of moles by the calculated volume of the solution. The latter was calculated according to the mass of H_2O or D_2O solution that was added, corrected for 1) the density of D_2O (1.11 g/ml, for solutions in D_2O), and 2) the solutions' volume increase upon the addition of the sugar, as described in Table S5.

Formulation #1A: [$^{13}C_6$, D_7]Glc in D_2O

202 mg of [$^{13}C_6$, D_7]Glc were combined with 289 mg of a D_2O solution containing 20.0 mM of OX063 and 1.3 mM of Gd^{3+}. The final concentrations in this formulation were 14 mM of OX063, 0.91 mM of Gd^{3+}, and 2.1 μmol of [$^{13}C_6D_7$]Glc per mg formulation (2.8 M).

Formulation #1B: [$^{13}C_6$]Glc in D_2O

196 mg of [$^{13}C_6$]Glc were combined with 245 mg of a D_2O solution containing 20 mM of OX063 and 1.3 mM of Gd^{3+}. The final concentrations in this formulation were 13.3 mM of OX063, 0.87 mM of Gd^{3+}, and 2.4 μmol of [$^{13}C_6$]Glc per mg formulation (3.2 M).

Formulation#2A: [$^{13}C_6$, D_8]2DG in D_2O

234 mg of [$^{13}C_6$, D_8]2DG were combined and with 335 mg of D_2O solution containing 20 mM of OX063 and 1.3 mM of Gd^{3+}. The final concentrations in this formulation were 14 mM OX063, 0.91 mM Gd^{3+} and 2.3 μmol of [$^{13}C_6D_8$]2DG per mg formulation (3.0 M).

Formulation #2B: [$^{13}C_6$]2DG in D_2O

159 mg of [$^{13}C_6$]2DG were combined with 227 mg of a D_2O solution containing 20 mM of OX063 and 1.3 mM of Gd^{3+}. The final concentrations in this formulation were 14 mM of OX063, 0.91 mM of Gd^{3+}, and 2.4 μmol of [$^{13}C_6$]2DG per mg formulation (3.2 M).

Typical formulations for the ^{15}N-labeled compounds are described below and in Table S1.

Formulation #3A: [$^{15}N_2$]urea in D_2O:glycerol

70.9 mg of [$^{15}N_2$]urea were combined with 186.6 mg of a 60:40 D_2O:glycerol solution and 3.4 mg of OX063. The final concentrations in this formulation were 14.9 mM OX063 and 4.38 μmol of [$^{15}N_2$]urea per mg formulation.

Formulation #3B: [$^{15}N_2$]urea in H_2O:glycerol

70.9 mg of [$^{15}N_2$]urea were combined with 186.6 mg of a 60:40 H_2O:glycerol solution and 3.4 mg of OX063. The final concentrations in this formulation were 14.1 mM OX063 and 4.38 μmol of [$^{15}N_2$]urea per mg formulation.

Formulation #4A: sodium [^{15}N]nitrate in D_2O:glycerol

210.4 mg of a D_2O solution containing 7.5 M of [^{15}N]nitrate were combined with 64.6 mg of glycerol and 4.3 mg of OX063. The final concentrations in this formulation were 12.5 mM OX063 and 5.09 μmol of [^{15}N]nitrate per mg formulation.

Formulation # 4B: sodium [^{15}N]nitrate in H_2O:glycerol

210.4 mg of a H_2O solution containing 7.5 M of [^{15}N]nitrate was combined with 64.6 mg of glycerol and 4.3 mg of OX063. The final concentrations in this formulation were 11.5 mM OX063 and 5.65 μmol of [^{15}N]nitrate per mg formulation.

Data analysis for polarization buildup

For ^{13}C polarization, the data were obtained using the polarizer's internal software. For ^{15}N polarization, each frequency domain spectrum was analyzed using a single Lorentzian line fitting and integration in Matlab (Mathworks, Natick, MA, USA). The polarization buildup time courses were fitted using the curve fitting option in Matlab, using the polarization buildup equation (Eq. 1), where $P_{(t)}$ is the polarization level at each time point, P_{max} is the maximal polarization level that could be reached, and T_b is the polarization buildup time constant.

$$P_t = P_{max}\left(1 - e^{-\frac{t}{T_b}}\right) \qquad \text{Eq. 1}$$

Table S1. Formulations' components

Stable isotope labeled sugar	Formulation #	Sugar (mg)	Total H_2O or D_2O added (mg)	Sugar / solution ratio	Sugar concentration (M)	OX063 final concentration (mM)	Gd^{3+} final Concentration (mM)	D_2O / H_2O
[$^{13}C_6$, D_7]Glc	1A	202	289	0.70	2.8	14.0	0.91	D_2O
	1C	195	251	0.70	2.8	13.9	0.91	H_2O
[$^{13}C_6$]Glc	1B	196	245	0.80	3.2	13.3	0.87	D_2O
[$^{13}C_6$, D_8]2DG	2A	234	335	0.70	3.0	14.0	0.91	D_2O
[$^{13}C_6$]2DG	2B	159	227	0.70	3.2	14.0	0.91	D_2O
^{15}N-labeled compound	Formulation #	^{15}N-labeled compound (mg)	Total H_2O:glycerol or D_2O:glycerol added (mg)*	^{15}N-labeled compound / solution ratio		OX063 final concentration (mM)		D_2O / H_2O with glycerol
[$^{15}N_2$]urea	3A	70.9	186.6	0.38		14.9		D_2O
[$^{15}N_2$]urea	3B	70.9	186.6	0.38		14.1		H_2O
Sodium [^{15}N]nitrate	4A	88.1	275	0.32		12.5		D_2O
Sodium [^{15}N]nitrate	4B	97.8	275	0.36		11.5		H_2O

About 480-600 mg of each formulation were placed in the polarizer cup for monitoring MW irradiation profiles, and for recording the polarization buildup of [$^{13}C_6$]2DG and [$^{13}C_6$]Glc (formulations #1B and 2B). For recording the polarization buildup of [^{15}N]urea and sodium [^{15}N]nitrate approximately 110-120 mg and 190-200 mg of each formulation was used, respectively.

Table S2. Conditions and characteristics of deuterated and non-deuterated sugars' polarization.

Formulation	Temperature (K)	Buildup time constant (min)	Maximal polarization level (a.u.)	Weight of formulation in the cup (mg)	µmol/ mg	µmol in the cup	Maximal polarization level normalized to µmol sugar in the cup
[$^{13}C_6$,D_7]Glc in D_2O #1A	1.5	22	2,927	2,927	2.1	1,023	286
[$^{13}C_6$,D_7]Glc in D_2O #1A	1.5	26	3,489	3,489	2.1	938	372
[$^{13}C_6$,D_7]Glc in D_2O #1A	1.5	20	2,053	2,053	2.1	853	241
[$^{13}C_6$,D_7]Glc in D_2O #1A	1.5	23	292	292	2.1	82	358
[$^{13}C_6$,D_7]Glc in D_2O #1A	1.5	27	484	484	2.1	84	577
[$^{13}C_6$,D_7]Glc in D_2O #1A	1.5	18	441	441	2.1	85	522
[$^{13}C_6$,D_7]Glc in D_2O #1A	1.5	30	403	403	2.1	86	469
Average ± standard deviation		**24 ± 4**		Average ± standard deviation			**403 ± 124**
[$^{13}C_6$,D_7]Glc in H_2O #1C	1.5	22	3,529	442	2.3	1,017	347
[$^{13}C_6$,D_7]Glc in H_2O #1C	1.5	23	3,947	418	2.3	961	411
[$^{13}C_6$,D_7]Glc in H_2O #1C	1.5	20	2,293	394	2.3	906	253
Average ± standard deviation		**22 ± 2**		Average ± standard deviation			**337 ± 79**
[$^{13}C_6$]Glc in D_2O #1B	1.5	27	207	438	2.4	1,051	20
[$^{13}C_6$]Glc in D_2O #1B	1.5	5	283	430	2.4	1,032	27
[$^{13}C_6$]Glc in D_2O #1B	1.5	7	215	422	2.4	1,012	21
Average ± standard deviation		**13 ± 12**		Average ± standard deviation			**23 ± 4**
[$^{13}C_6$,D_8]2DG in D_2O #2A	1.5	22	3,391	564	2.3	1,297	261
[$^{13}C_6$,D_8]2DG in D_2O #2A	1.5	22	3,397	532	2.3	1,223	278
[$^{13}C_6$,D_8]2DG in D_2O #2A	1.5	21	2,515	500	2.3	1,149	219
Average ± standard deviation		**22 ± 1**		Average ± standard deviation			**253 ± 30**
[$^{13}C_6$]2DG in D_2O #2B	1.5	17	346	384	2.4	922	38
[$^{13}C_6$]2DG in D_2O #2B	1.5	10	390	360	2.4	864	45
[$^{13}C_6$]2DG in D_2O #2B	1.5	7	305	336	2.4	806	38
Average ± standard deviation		**11 ± 5**		Average ± standard deviation			**40 ± 4**

Table S3. Chronological order of ^{13}C MW sweep measurements for the sugar formulations.

Chronological order of experimental measurements*	Formulation	Sample weight (mg)
I	1A	487
II	1A	487
III	1C	442
IV	1B	438
V	2B	384
VI	2A	564

All studies were performed on different days.

Table S4. Chronological order of ^{3}C polarization buildup measurements for the sugar formulations.

Chronological order of experimental measurements	Formulation	Sample weight (mg)
1	1A	487
2	1C	442
3	1B	438
4	2B	384
5	2A	564
6	1A	487
7	1C	442
8	1B	438
9	2B	384
10	2A	564
11	1A	487
12	1C	442
13	1B	438
14	2B	384
15	2A	564
16	1A	40.9
17	1A	40.3
18	1A	40.0
19	1A	38.9

Table S5. Volume increase in mixtures of glucose and water which were used in the formulations.

Mixture number	Volume of H_2O used in mixture test (mL)	Weight of glucose used in mixture test (g)	Glucose-to-water ratio (g/g)	Final volume of mixture (mL)	Factor of volume increase
1	5.00	4.000	0.8	7.50	1.50
2	5.00	3.505	0.7	7.15	1.43
3	5.00	2.992	0.6	6.84	1.37

* All mixtures were prepared and tested at room temperature (about 20 °C), using DDW and naturally abundant D-glucose.

Table S6. The various conditions of protonation and deuteration of X-nuclei tested in this work.

Compound	Proton binding site available?	Proton binding site protonated?	Proton binding site deuterated?	Enhancement of maximal polarization due to deuteration of proton binding sites or bath
[$^{13}C_6$, D_7]Glc	Yes	No	Yes	17.5
[$^{13}C_6$]Glc	Yes	Yes	No	
[$^{13}C_6$,D_8]2DG	Yes	No	Yes	6.3
[$^{13}C_6$]2DG	Yes	Yes	No	
[$^{15}N_2$]urea in D_2O:glycerol	Yes	No/Partly	Yes/Partly	2.2
[$^{15}N_2$]urea in H_2O:glycerol	Yes	Yes	No	
Sodium [$^{15}N_2$]nitrate in D_2O:glycerol	No	NA	NA	None
Sodium [$^{15}N_2$]nitrate in H_2O:glycerol	No	NA	NA	

NA – not applicable.

Table S7. T_1 relaxation times in solution

Compound	Nucleus	T_1 (s)	TE / HP	Concentration of agent and osmolarity	Temp.	Magnetic Field	Publication
[1-^{13}C]D-glucose	^{13}C	2	HP		37 °C	14T	Harada *et al.* 2010[1]
[$^{13}C_6$,D_7]D-glucose	^{13}C-$C_1\alpha$	13.4	TE	40 mM, 400 mOsm	37 °C	7T	Allouche-Arnon *et al.* 2013[2]
	^{13}C-$C_1\beta$	13.5					
	^{13}C-(C_2+ C_5)α	13.6					
	^{13}C-$C_2\beta$	12.7					
	^{13}C-$C_3\alpha$	12.5					
	^{13}C-C_4(α + β)	11.9					
	^{13}C-(C_3+ C_5) β	12.9					
	^{13}C-C_6(α + β)	9.7					
	^{13}C-all	9.5	HP	26 mM, 314 mOsm	20 °C	3T	Allouche-Arnon *et al.* 2013[2]
	^{13}C-$C_1\beta$	12.0	HP	11 mM, 300 mOsm	21 °C	5.8T	Sapir *et al.* 2019[3]
	^{13}C-$C_1\alpha$	12.2					
	^{13}C-C_6	9.3					
	^{13}C-$C_1\beta$	16.9			40 °C		
	^{13}C-$C_1\alpha$	16.8					
	^{13}C-C_6	12.3					
[$^{13}C_6$,D_8]2-deoxy-D-glucose	^{13}C-$C_1\beta$	14.3			21 °C		
	^{13}C-$C_1\alpha$	14.3					
	^{13}C-C_6	10.2					
	^{13}C-$C_2\beta$	9.8					
	^{13}C-$C_2\alpha$	9.8					
	^{13}C-$C_1\beta$	24.0			40 °C		
	^{13}C-$C_1\alpha$	23.5					
	^{13}C-C_6	16.7					
	^{13}C-$C_2\beta$	15.9					
	^{13}C-$C_2\alpha$	15.2					
[$^{15}N_2$,D_4]urea ([$^{15}N_2$]urea dissolved in D_2O)	^{15}N	146	HP		15-25 °C	5.8T	Harris *et al.* [4]
		191			25-35 °C		
		226			35-45 °C		
		308			45-55 °C		
		351			55-65 °C		
		382			65-75 °C		
[$^{15}N_2$]urea ([$^{15}N_2$]urea dissolved in H_2O)	^{15}N	33	HP		35-40 °C		
Sodium [^{15}N]nitrate	^{15}N	172	HP	19-29 mM, D_2O	10-19 °C	5.8T	Gamliel *et al.* [5]
		139			20-23 °C		
		109			34-44 °C		
		105			40-50 °C		
		98		19-29 mM, H_2O	33-44 °C		
		102		19-29 mM, saline	33-44 °C		

TE, Thermal equilibrium; HP, Hyperpolarized; Temp., temperature; RT, room temperature.

Figure S1. MW frequency sweep profiles of deuterated and non-deuterated ^{13}C-labeled sugars in H_2O and in D_2O.

The first maxima of the sugar formulations used in the current study were found to be the same and this frequency was used for recording the polarization buildup time courses.

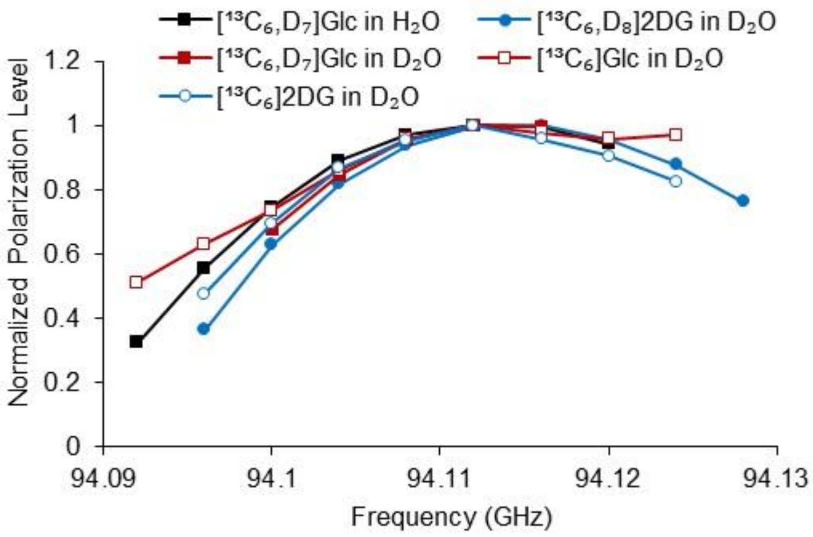

The data were normalized to the highest point of each profile. Data are presented as obtained from the polarizer's spectrometer, in magnitude mode.

Figure S2. Individual time courses and curve fitting for polarization buildup of deuterated and non-deuterated ^{13}C-uniformly-labeled sugars.

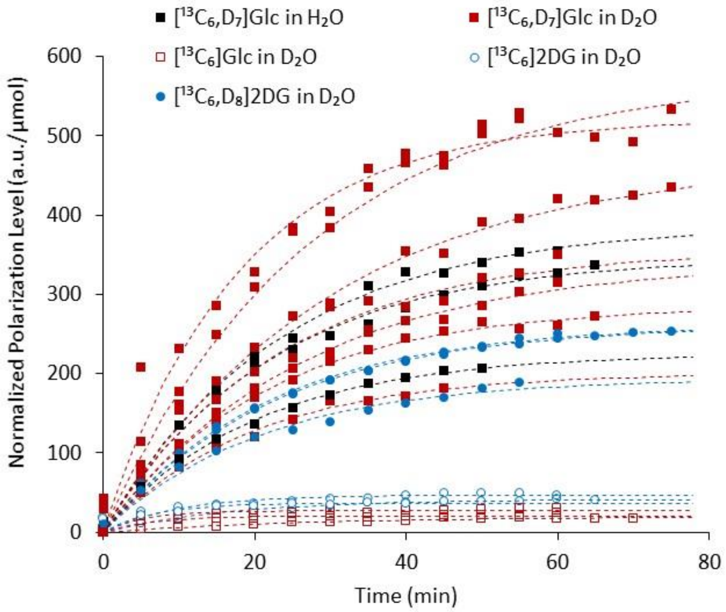

The actual polarization levels in arbitrary units for each time course were corrected for the number of sugar moles in the cup. The dotted line was plotted using the buildup time constant and maximal polarization level that resulted from curve fitting of each experimental time course to Eq. 1.

Figure S3. Example intensity profiles of MW sweeps obtained with the formulations containing ^{15}N in D_2O:glycerol.

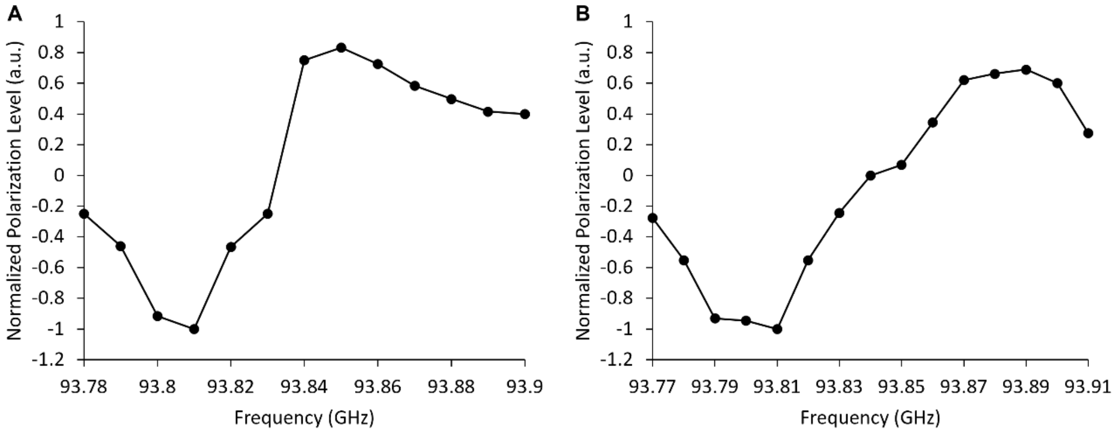

The data were normalized to the lowest point of each profile. A) [$^{15}N_2$]urea, B) sodium [^{15}N]nitrate.

Figure S4. Individual polarization buildup data for ^{15}N-labeled agents.

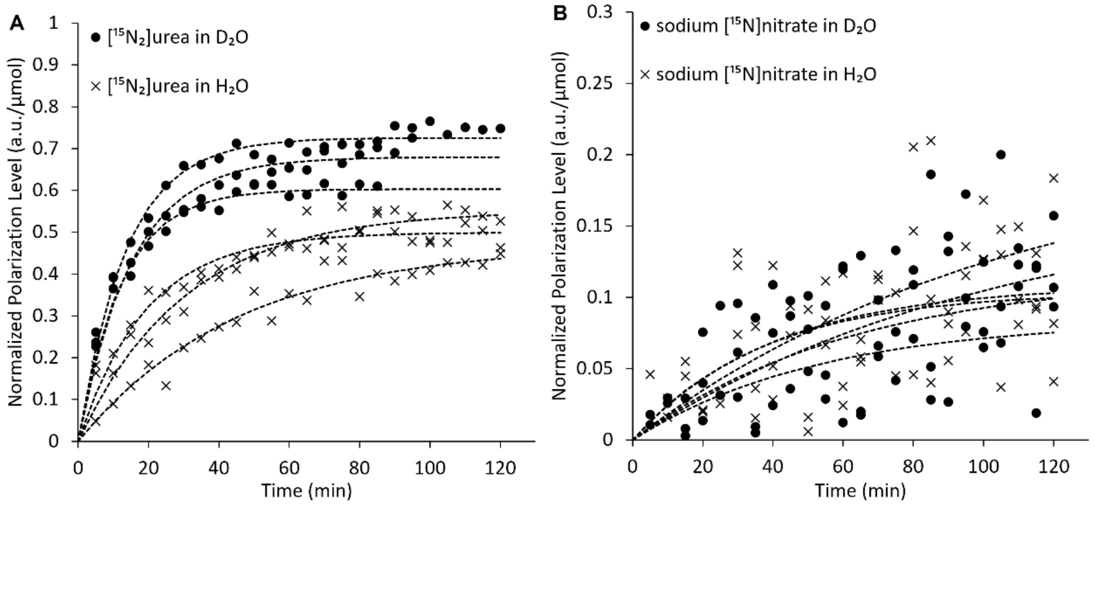

Figure S5. Solid-state polarization buildup and decay of ^{13}C-labeled 2DG with or without deuteration.

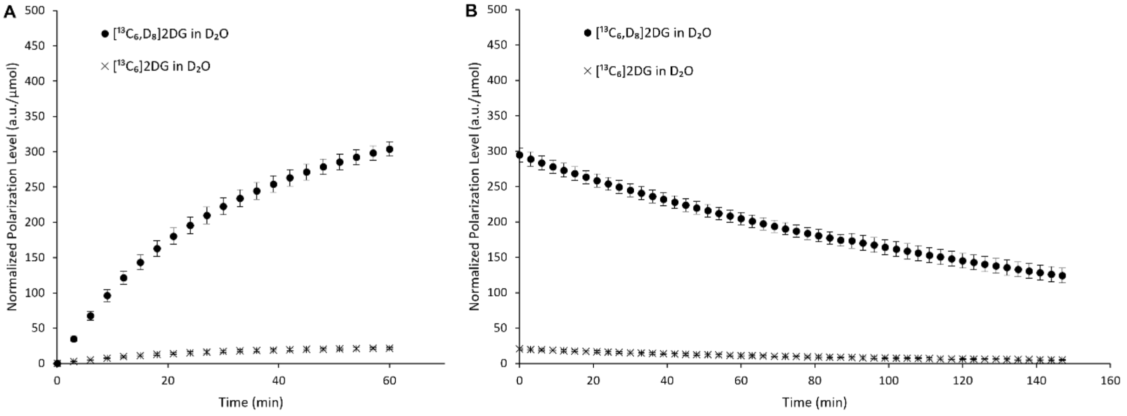

Further to obtaining higher polarization levels for the X-nuclei which were directly bound to deuterons, we wished to explore the potential mechanism underlying this observation. A possible explanation has to do with prolongation of the solid-state T_1, which would allow favorable buildup conditions. To this end, the experiments described in Figure 1B and Table 1 with ^{13}C- and deuterium-labeled-2DG analogs were reproduced on a second Hypersense polarizer at the University of Oxford.

A) A reproduction of the experiments shown in Figure 1B, with the same samples used for producing Figure 1B (n=3, for each sample, Formulations 2A and 2B).

B) At the end of each buildup duration, the MW irradiation was stopped, and the polarization level was monitored during its decay.

M_z was calculated from the M_{xy} data shown in this plot using the conversion: $M_{xy}(t)=M_z(t)\cdot\sin(\theta)$, where θ, the flip angle for excitation, was 5 °. The decay of $M_z(t)$ data was then used to calculate the T_1 of $[^{13}C_6,D_8]$2DG and $[^{13}C_6]$2DG in solid-state.

The solid-state T_1 of $[^{13}C_6,D_8]$2DG was found to be 1.8-fold longer than that of $[^{13}C_6]$2DG (185.7± 36.2 min and 101.7± 32.5 min, respectively). In this set of experiments the increase in maximal polarization level of the deuterated compound was 14.8-fold (more than in the previous set of experiments, Table 1) and the buildup time constant increased 1.2-fold (less

than in the previous set of experiments, Table 1). These differences are likely due to slightly different temperatures of the sample during the DNP process across the two spin polarization systems, as it was previously shown that such sugar molecules' polarization is very sensitive to the temperature of the sample during the DNP process [6]. Nevertheless, the higher polarization level of the ^{13}C sites directly bound to deuterons was reproduced and the T_1 in solid-state was indeed prolonged, providing a possible explanation for this observation.

References

1. Harada M, Kubo H, Abe T, Maezawa H, Otsuka H. Selection of endogenous C-13 substrates for observation of intracellular metabolism using the dynamic nuclear polarization technique. *Japanese Journal of Radiology.* 2010;28(2):173-179.
2. Allouche-Arnon H, Wade T, Waldner LF, et al. In vivo magnetic resonance imaging of glucose - initial experience. *Contrast Media Mol Imaging.* 2013;8(1):72-82.
3. Sapir G, Harris T, Uppala S, et al. C-13(6),D-8 2-deoxyglucose phosphorylation by hexokinase shows selectivity for the beta-anomer. *Sci Rep.* 2019;9.
4. Harris T, Gamliel A, Uppala S, et al. Long-lived N-15 hyperpolarization and rapid relaxation as a potential basis for repeated first pass perfusion imaging - marked effects of deuteration and temperature. *ChemPhysChem.* 2018;19(17):2148-2152.
5. Gamliel A, Uppala S, Sapir G, et al. Hyperpolarized N-15 nitrate as a potential long lived hyperpolarized contrast agent for MRI. *J Magn Reson.* 2019;299:188-195.
6. Harris T, Gamliel A, Nardi-Schreiber A, Sosna J, Gomori JM, Katz-Brull R. The effect of Gadolinium doping in C-13(6),H-2(7) glucose formulations on C-13 dynamic nuclear polarization at 3.35 T. *ChemPhysChem.* 2020.
